# KODAMA exploratory analysis in metabolic phenotyping

**DOI:** 10.3389/fmolb.2022.1070394

**Published:** 2023-01-17

**Authors:** Maria Mgella Zinga, Ebtesam Abdel-Shafy, Tadele Melak, Alessia Vignoli, Silvano Piazza, Luiz Fernando Zerbini, Leonardo Tenori, Stefano Cacciatore

**Affiliations:** ^1^ Bioinformatics Unit, International Centre for Genetic Engineering and Biotechnology, Cape Town, South Africa; ^2^ Department of Medical Parasitology and Entomology, Catholic University of Health and Allied Sciences, Mwanza, Tanzania; ^3^ National Research Centre, Cairo, Egypt; ^4^ Computation Biology, International Centre for Genetic Engineering and Biotechnology, Trieste, Italy; ^5^ Department of clinical chemistry, University of Gondar, Gondar, Ethiopia; ^6^ Magnetic Resonance Center (CERM) and Department of Chemistry “Ugo Schiff”, University of Florence, Sesto Fiorentino, Italy; ^7^ Consorzio Interuniversitario Risonanze Magnetiche Metallo Proteine (CIRMMP), Sesto Fiorentino, Italy; ^8^ Cancer Genomics, International Centre for Genetic Engineering and Biotechnology, Cape Town, South Africa; ^9^ Institute of Reproductive and Developmental Biology, Imperial College London, London, United Kingdom

**Keywords:** KODAMA, unsupervised, semi-supervised, metabolomics, clustering

## Abstract

KODAMA is a valuable tool in metabolomics research to perform exploratory analysis. The advanced analytical technologies commonly used for metabolic phenotyping, mass spectrometry, and nuclear magnetic resonance spectroscopy push out a bunch of high-dimensional data. These complex datasets necessitate tailored statistical analysis able to highlight potentially interesting patterns from a noisy background. Hence, the visualization of metabolomics data for exploratory analysis revolves around dimensionality reduction. KODAMA excels at revealing local structures in high-dimensional data, such as metabolomics data. KODAMA has a high capacity to detect different underlying relationships in experimental datasets and correlate extracted features with accompanying metadata. Here, we describe the main application of KODAMA exploratory analysis in metabolomics research.

## 1 Introduction

Metabolomics is the discipline that involves systematic profiling and analysis of metabolites and their fluctuations (Vignoli et al., 2021). Metabolomics has been applied to many fields of research, including studies in non-communicable and infectious diseases. (Cacciatore and Loda, 2015; Vignoli et al., 2020; Bataineh et al., 2022), molecular biology (Semreen et al., 2020; AL Bataineh et al., 2021), and food research (Maccaferri et al., 2012; Ojo-Okunola et al., 2020). In the medical field, it has played a key role in enhancing research in personalized medicine (Cacciatore et al., 2018). Nuclear magnetic resonance (NMR) spectroscopy and mass spectrometry (MS) are the major platforms used to provide structural and quantitative information on metabolites in biological samples (Lenz and Wilson, 2007; Lindon et al., 2011; McCartney et al., 2019; Takis et al., 2019; Vignoli et al., 2019). A variety of metabolomics databases are created to store structural and quantitative information from these platforms. The Human Metabolome Database (HMDB) (Wishart et al., 2018) and the LIPID MAPS Structure Database (LMSD) (Sud et al., 2007) are among the commonest metabolomics databases.

Powerful analysis techniques and software tools are needed to address the large amount and variety of data generated by these platforms (Camacho et al., 2018). Advances in artificial intelligence, including machine learning (ML), have contributed to breakthroughs in different scientific disciplines through discovery and innovations in clinical and biological research (Rajkomar et al., 2019).

**FIGURE 1:**
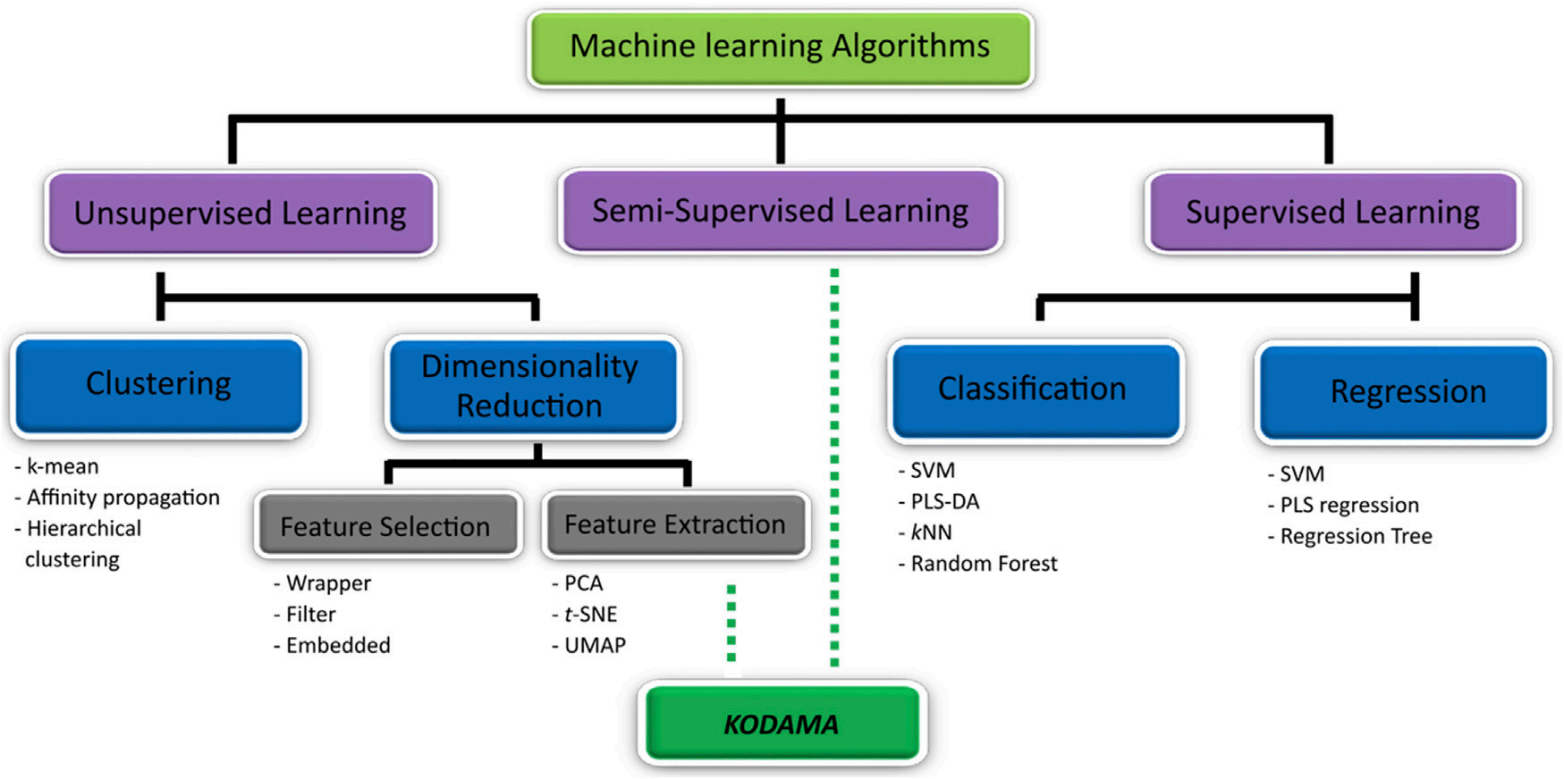
KODAMA on machine learning algorithm’s map. The machine learning algorithm can be categorized in unsupervised (i.e., clustering and dimensionality reduction method) and supervised learning. KODAMA is one of unsupervised learning methods used for dimensionality reduction. Optionally, if supervised information is used to lead the process of discovery of new patterns, KODAMA can be classified as semi-supervised.

ML methods are employed in metabolomics (Figure 1) in the process of building predictive models (supervised learning) or identifying informative groupings within data (unsupervised learning) (Greener et al., 2022). Supervised learning algorithms predict the class (classification) or value (regression) of unlabeled datasets using a model based on a predefined set of data points and associated information (i.e., class or value) (Berry et al., 2019). Among the supervised learning algorithm, *k*-nearest neighbors (*k*NN) (Romano et al., 2018; Romano et al., 2019; Di Donato et al., 2021), partial least squares (PLS) (Bertini et al., 2012; Vignoli et al., 2022) and its variant orthogonal PLS (O-PLS) (Cacciatore et al., 2017b), support vector machine (SVM) (Cacciatore et al., 2013; Paglia et al., 2016), and random forest (RF) (Tenori et al., 2015; McCartney et al., 2019) are the most used techniques in metabolomics research. One of the performance metrics used to assess the quality of prediction is cross-validated accuracy. Briefly, a dataset is separated into training and test sets, where a predictor is built on the training set to predict the class or the values of the samples in the test set. This process is repeated multiple times with different combinations of training and test sets to calculate an average of model performances (cross-validated accuracy).

On the other hand, unsupervised learning aims to identify unknown data patterns without prior existing knowledge of groupings within a dataset. Methods belonging to this category include clustering algorithms (e.g., *k*-means and hierarchical clustering) and dimensionality reduction methods (Berry et al., 2019). Clustering refers to the identification of groups within the dataset using algorithms to determine similarities which allow data points to be grouped into subsections and patterns within the dataset (Ren et al., 2015). Dimensionality reduction methods transform data with high dimensionality (many variables) into data of lesser dimensions while minimizing the loss of information. These methods can be distinct in feature selection or feature extraction. However, feature selection methods, such as univariate filter, wrapper, and embedded methods, aim to select a subset of the features that best explains the original dataset; feature extraction methods extract new features on the basis of combinations of the original features (Hira and Gillies, 2015).

Principal component analysis (PCA) is the most used feature extraction method in metabolomics (Blekherman et al., 2011; Hendriks et al., 2011; Saccenti et al., 2014). It reduces the dimensionality of the dataset while preserving variability by finding new variables that are linear functions of the ones in the original dataset, thereby maximizing variance (Pearson, 1901; Sewell, 2007). Despite the wide integration in various analyses, PCA shows inefficient performance for dimensionality reduction on large datasets (Yang et al., 2021). It failed to extract features from non-linear data and does not maintain the local structure of the data when the size of the dimension increases. In many cases, the complexity of the datasets requires the use of more flexible solutions to highlight interesting patterns in the data. Methods, such as *t*-distributed stochastic neighbor embedding (*t*-SNE) (Van der Maaten and Hinton, 2008) and uniform manifold approximation and projection (UMAP) (McInnes et al., 2018), which have seen their popularity grow in the analysis of a large dataset through single-cell RNA sequencing, have been recently applied to the analysis of a large metabolomic dataset (Buergel et al., 2022). They have the advantages of maintaining neighbor information and visualizing the local structure (Becht et al., 2019; Yang et al., 2021). Although the debate is focused on the advantages and disadvantages of using *t*-SNE or UMAP in terms of the global structure of the data, little attention is dedicated to their sensitivity to the noise, typical in biological datasets.

**FIGURE 2:**
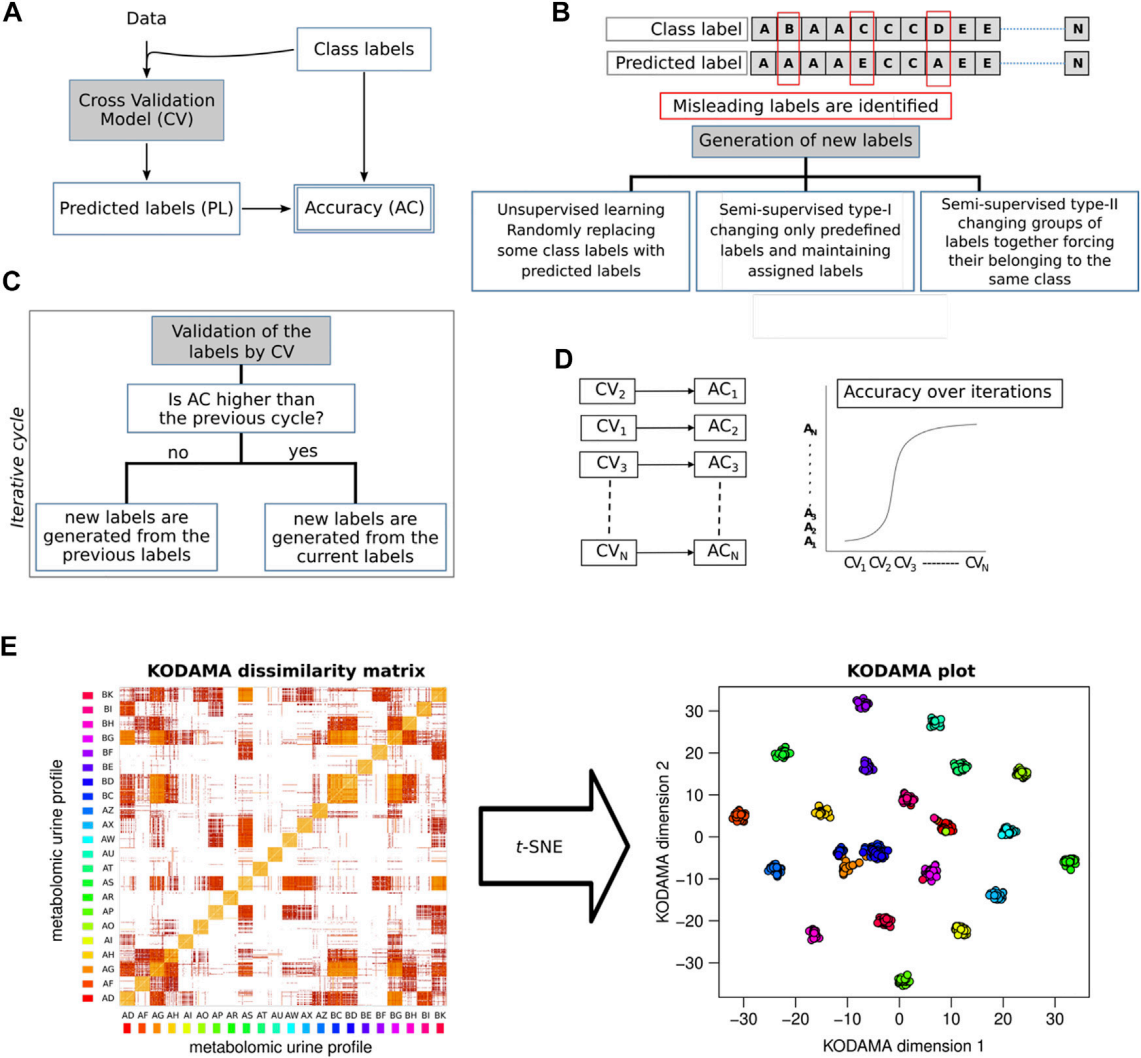
KODAMA accuracy maximization by iterative cross-validations. **(A)** Cross-validation model (CV) generates predicted labels (PLs) that are used to calculate the accuracy value (AC). **(B)** Generation of new labels to conduct the process of accuracy maximization can be i) an unsupervised method, randomly swapping some class labels of misleading samples with predicted labels; ii) semi-supervised type-I, changing only predefined labels and maintaining assigned class labels; or iii) semi-supervised type-II, changing groups of labels together forcing their belonging to the same class. **(C)** Generation of new labels is an iterative process aimed to identify the labels with the highest cross-validated accuracy. **(D)** Accuracy values increase with the number of iterations. **(E)** KODAMA dissimilarity matrix generated as output can be transformed with MDS, or *t*-SNE, in a low-dimensional space.

In this review, we will focus on KODAMA, an unsupervised machine-learning algorithm for feature extraction from noisy and high-dimensional data (Cacciatore et al., 2014). Unlike other methods, KODAMA results are driven by an integrated procedure of cross-validation of the results (Figures 2A–D). Golub et al. (1999) showed that a predictor based on clustering can be refined, removing samples not correctly predicted in cross-validation. We introduced the novel idea that a clustering itself can be improved by editing the class labels of samples not correctly predicted in cross-validation. In the core step of KODAMA, an initial clustering is refined through an iterative procedure, aiming to maximize the cross-validated accuracy by swapping the class labels of not correctly predicted samples with their predicted class value. The initial clustering can either be the result of any clustering methods or simply a vector where each sample belongs to a different class. In the current version, the cross-validated accuracy can be calculated by using *k*NN or PLS. The iterative procedure used in KODAMA leads to suboptimal solutions and is repeated to average the effects, owing to randomness. After each run of the procedure, a classification vector with high cross-validated accuracy is obtained. KODAMA subsequently collects and processes these results by constructing a dissimilarity matrix to provide a holistic view of the data while maintaining their intrinsic structure. The KODAMA dissimilarity matrix can be visualized in a low-dimensional space (generally in two dimensions) using methods, such as multidimensional scaling (MDS), where the pair-wise dissimilarity and similarity between samples are preserved (Figure 2E). The final output could be visualized as a set of points in a Cartesian space with a low number of dimensions (KODAMA dimensions).

The algorithm is freely available from the R archive CRAN (http://cran.r-project.org) and included as a function in the homonym package (Cacciatore et al., 2017a). Since version 2.0 of the KODAMA package, t-SNE can be used to transform the dissimilarity matrix in low-dimensional space instead MDS.

This versatile method has been successfully applied to other disciplines including genomics (Meucci et al., 2019). Here, we will introduce the KODAMA application in metabolomics research.

## 2 Integration with clustering algorithms

Clustering methods are common techniques used in exploratory data analysis to group together observations in different subsets, where observations in one subset are more similar to each other than observations in different subsets. Results can depend on the chosen method’s assumptions and starting parameter values. There is a wide array of clustering approaches, each with its strengths and weaknesses. Hierarchical clustering and partition clustering are the two classes of clustering algorithms mostly used in biological research.

The ability of KODAMA in highlighting local structures facilitates the identification of clusters. The benefit of using clustering on the KODAMA dimensions was shown using simulated and experimental datasets and comparing the results of different clustering methods with KODAMA that showed a clear separation of classes (Cacciatore et al., 2014). Both partitional and hierarchical clustering can be applied to the KODAMA dimensions, as shown in different metabolomic studies described in the following paragraphs.

Partitional clustering methods, such as partition around medoids (PAM) clustering, were applied to the KODAMA dimensions to identify different phenotypes in a dataset of urine metabolome of women with lower urinary tract symptoms (Bray et al., 2017) and in a dataset of lipoprotein profiles of patients with pancreatic ductal adenocarcinoma (Elebo et al., 2021). Hierarchical clustering algorithms are largely used for the visualization of metabolic data through heatmap plots. Hierarchical clustering was also successfully applied to the output of KODAMA to identify metabolic phenotypes in the plasma of patients with prostate cancer (Cacciatore et al., 2021) and visualize metabolic data for MYC- and AKT-driven prostate cancer (Priolo et al., 2014).

In general, determining the number of clusters that fit a certain dataset is required to apply a partitional clustering or to perform a “tree cutting” of the hierarchical clustering’s dendrogram. The silhouette algorithm is one of the methods used to determine the optimal number of clusters. It computes the coefficients of each point from the measure of how much that point is similar to its own cluster compared to other clusters. The silhouette algorithm has been used to determine the optimal number of clusters both in PAM (Elebo et al., 2021) and hierarchical clustering (Cacciatore et al., 2021) on the KODAMA score. Identification of the number of clusters has shown their benefit when applied to the analysis of KODAMA scores (Cacciatore et al., 2017a).

## 3 KODAMA exploratory analysis in metabolomics research

Feature extraction facilitates the classification, visualization, and communication of high-dimensional data such as the those generated by omics sciences, including metabolomics (Hinton and Salakhutdinov, 2006). Unsupervised approaches are particularly useful to exploratively identify clustering patterns in the data and in metabolomic research. Previous studies harnessed the KODAMA algorithm to identify the metabolic phenotype in various disciplines: psychiatric, oncologic, and pregnancy research.

### 3.1 Psychiatry

The identification of early biomarkers of psychotic experiences (PEs) is pivotal to timely diagnosis and effective treatment of patients at risk of future disorders, improving clinical outcomes and life quality, particularly in children and adolescents (Larsen et al., 2011). Madrid-Gambin et al. (2019) performed an integrated plasma lipidomic and proteomic study on a population of 115 children (48 cases and 67 controls) aimed at identifying early metabolic biomarkers of PEs. All patients were prospectively enrolled and evaluated, and plasma samples were collected at 12 years of age and re-evaluated at 18 years of age to identify those with definite PEs. The univariate analysis enabled the identification of a panel of 16 lipids, and one protein significantly dysregulated in children with PEs, as compared to controls. The KODAMA algorithm was used to identify potential underlying metabolic phenotypes in the study population: according to the highest silhouette median values, four clusters emerged. PE occurrence was significantly different among the four clusters. Particularly, as compared with all the others, the cluster named D, characterized by increased levels of small LDL particles, represents a metabolic phenotype with a high probability of developing PEs (occurrence 71%). The results of this study suggest early vulnerability to the development of PEs could have a metabolic basis in which the lipidome plays a key role.

### 3.2 Oncology

The KODAMA algorithm has found its way into oncological metabolomic research. Prostate cancer (PC) is the second most frequently diagnosed cancer in men and the Black population, as compared to the other ethnicities, and has a higher risk of developing particularly aggressive PCs. Cacciatore et al. (2021) analyzed *via* NMR plasma samples of 41 South African men diagnosed with PC. Glycoproteins (GlycA and GlycB), well-known metabolic markers of systemic inflammation, were found to be significantly higher in patients with highly aggressive and high-stage (metastatic) diseases. Moreover, GlycA and GlycB showed significant correlations with the prostate-specific antigen. Interestingly, KODAMA enabled the identification of four metabolic clusters associated with PC aggressiveness. The metabotype IV, characterized by high levels of GlycA and GlycB, is the one associated with the worst oncological condition (and outcome), and it can be discriminated from all the others with high accuracy (PLS model accuracy: 91.2%). If further validated, the metabotype IV represents a well-defined high-risk metabolomic profile that, in future, could be used to predict patients who will be more likely to benefit from combination therapy that associates androgen deprivation with drugs that are able to reduce the level of systemic inflammation.

Elebo et al. (2021) conducted a pilot serum NMR-based metabolomic and lipoproteomic study on 34 patients diagnosed with pancreatic ductal adenocarcinoma (PDAC), 6 patients with chronic pancreatitis, and 6 healthy participants. In this study, KODAMA highlighted three distinct clusters: all healthy controls and patients with chronic pancreatitis were allocated in the cluster named N, whereas PDAC patients of clusters A and B were characterized by higher free cholesterol and cholesterol ester ratio (ratio >.45). Moreover, patients clustered in A and B, as compared to those in the cluster N, displayed a significant dysregulation of liver function parameters. The A–B profiles could represent the patient’s phenotype of patients at a high risk of obstructive jaundice that may require urgent treatment.

### 3.3 Pregnancy

The KODAMA algorithm was also applied to study the metabolic phenotyping of women with lower urinary tract symptoms (LUTS) (Bray et al., 2017). Urine samples of 176 women attending tertiary urogynecology clinics and 36 healthy control women attending general gynecology clinics were analyzed through NMR spectroscopy. Despite the high urine metabolic variability, KODAMA identified four distinct urinary metabotypes associated with the variations of six clinical parameters (i.e., BMI, parity, frequency, straining, storage score, and OAB status). In particular, the metabotypes 1 and 4 showed to be the most discriminated: Metabotype 1 was enriched in patients with increased BMI and decreased frequency, whereas the opposite trends were observed in metabotype 4 patients. Interestingly, hippurate and isoleucine were crucial in this discrimination and, thus, probably play a role in LUTS. The depiction of these sub-phenotypes in such heterogeneous disease like LUTS could pave the way for more tailored pharmacological treatments, improving patient outcomes.

## 4 New paradigms of KODAMA

### 4.1 Semi-supervised approach

Semi-supervised learning is an approach that falls between supervised and unsupervised learning. It can be defined as a machine learning approach where the learning procedure is led by external supervised information. The procedure of maximization of cross-validated accuracy can be led by supervised information making KODAMA, optionally, a semi-supervised method.

There are two different ways to lead the feature extraction algorithm of KODAMA with external information (Figure 2B). In the first approach (type-I), external information can be provided as belonging to a particular sample classification (e.g., healthy status). This information is provided partially for only some samples, and it is used to lead the maximization of the cross-validated accuracy without changing the class of these samples. This led to improved model reliability, especially with limited access to curated labeled data.

In the second approach (type-II), the learning procedure considers the samples, as organized in groups. For example, if the dataset encompasses replicates, constraints can be imposed, linking some samples in such a way that if one of them is changed, the linked ones must change in the same way; they are forced to belong to the same class. This will produce a solution where linked samples are forced to have a close distance in the KODAMA scores.

KODAMA was applied as a semi-supervised type-II in a dataset containing metabolomic data on urine samples from a cohort of 22 healthy donors, where each provided about 40 urine samples over the time course of approximately 2 months, for a total of 873 samples (Cacciatore et al., 2014). The information relative to the donors of the urine samples was provided to the KODAMA algorithm. If the unsupervised KODAMA clearly separated the urine of each donor, providing this additional information, KODAMA was able to highlight the separation based on sex, which was not previously provided.

### 4.2 Chemical structural similarity analysis

Initially, KODAMA was designed as an unsupervised method to facilitate the identification of patterns representing underlying groups on all samples in a dataset. Recently, KODAMA has been introduced as a method for investigating the chemical similarity between metabolites. In the procedure implemented in the R package MetChem, KODAMA uses the molecular structure of metabolites represented by the simplified molecular-input line-entry system (SMILES) (Weininger, 1988) to visualize the chemical similarity across metabolites in two-dimensional space. SMILES are converted into molecular fingerprints, encoding their structural characteristics as a vector (Bender and Brown, 2018). The distance between two metabolites is calculated using a distance method, such as the Tanimoto distance method (Chen and Reynolds, 2002), to produce a dissimilarity matrix. This dissimilarity matrix is then converted into a multi-dimensional space by MDS prior to being processed by KODAMA. In this way, KODAMA can offer the possibility to identify the class of metabolites structurally that may be representative of specific functions and interactions in a biological context.

## 5 Conclusion

KODAMA is an innovative approach that can be used for unsupervised and semi-supervised exploratory analyses of high-dimensional data for feature extraction and clustering of data points into groups based on underlying features. The application of this method has shown its benefit in the stratification of several medical conditions. Recently, a new application aimed at the identification of structural similarities among metabolites has been shown.
