# Corrigendum: KODAMA exploratory analysis in metabolic phenotyping

**DOI:** 10.3389/fmolb.2023.1165720

**Published:** 2023-03-10

**Authors:** Maria Mgella Zinga, Ebtesam A. Abdel-Shafy, Tadele Melak, Alessia Vignoli, Silvano Piazza, Luiz Fernando Zerbini, Leonardo Tenori, Stefano Cacciatore

**Affiliations:** ^1^ Bioinformatics Unit, International Centre for Genetic Engineering and Biotechnology, Cape Town, South Africa; ^2^ Department of Medical Parasitology and Entomology, Catholic University of Health and Allied Sciences, Mwanza, Tanzania; ^3^ National Research Centre, Cairo, Egypt; ^4^ Computation Biology, International Centre for Genetic Engineering and Biotechnology, Trieste, Italy; ^5^ Department of clinical chemistry, University of Gondar, Gondar, Ethiopia; ^6^ Magnetic Resonance Center (CERM) and Department of Chemistry “Ugo Schiff”, University of Florence, Sesto Fiorentino, Italy; ^7^ Consorzio Interuniversitario Risonanze Magnetiche Metallo Proteine (CIRMMP), Sesto Fiorentino, Italy; ^8^ Cancer Genomics, International Centre for Genetic Engineering and Biotechnology, Cape Town, South Africa; ^9^ Institute of Reproductive and Developmental Biology, Imperial College London, London, United Kingdom

**Keywords:** KODAMA, unsupervised, semi-supervised, metabolomics, clustering

In the published article, there was an error in **Affiliations** of Leonardo Tenori. Instead of “5,6”, it should be “6,7”.

The authors apologize for this error and state that this does not change the scientific conclusions of the article in any way. The original article has been updated.

